# Association between Bone Mineral Density of Femoral Neck and Geriatric Nutritional Risk Index in Rheumatoid Arthritis Patients Treated with Biological Disease-Modifying Anti-Rheumatic Drugs

**DOI:** 10.3390/nu10020234

**Published:** 2018-02-18

**Authors:** Hiroto Tokumoto, Hiroyuki Tominaga, Yoshiya Arishima, Go Jokoji, Masaki Akimoto, Hideo Ohtsubo, Eiji Taketomi, Nobuhiko Sunahara, Satoshi Nagano, Yasuhiro Ishidou, Setsuro Komiya, Takao Setoguchi

**Affiliations:** 1 Department of Orthopaedic Surgery, Graduate School of Medical and Dental Sciences, Kagoshima University, Kagoshima 890-8520, Japan; tokuhiro1984@hotmail.co.jp (H.Tok.); hiro-tom@m2.kufm.kagoshima-u.ac.jp (H.Tom.); ariyoshi71@gmail.com (Y.A.); jokojigo@yahoo.co.jp (G.J.); n-sunahara@kagoshima-med.jrc.or.jp (N.S.); naga@m2.kufm.kagoshima-u.ac.jp (S.N.); skomiya@m3.kufm.kagoshima-u.ac.jp (S.K.); 2 Department of Orthopaedic Surgery, Japanese Red Cross Kagoshima Hospital, Kagoshima 891-0133, Japan; eiji.taketomi@nifty.com; 3 Department of Hematology and Immunology, Graduate School of Medical and Dental Sciences, Kagoshima University, Kagoshima 890-8520, Japan; akimoto@m3.kufm.kagoshima-u.ac.jp; 4 Center for Rheumatic Diseases, Japanese Red Cross Kagoshima Hospital, Kagoshima 891-0133, Japan; h-otsubo@kagoshima-med.jrc.or.jp; 5 Department of Medical Joint Materials, Graduate School of Medical and Dental Sciences, Kagoshima University, Kagoshima 890-8520, Japan; ishidou@m2.kufm.kagoshima-u.ac.jp; 6 The Near-Future Locomotor Organ Medicine Creation Course (Kusunoki Kai), Graduate School of Medical and Dental Sciences, Kagoshima University, Kagoshima 890-8520, Japan

**Keywords:** rheumatoid arthritis (RA), geriatric nutritional risk index (GNRI), bone mineral density (BMD), biological disease-modifying, anti-rheumatic drugs (bDMARDs), osteoporosis

## Abstract

Treatment of rheumatoid arthritis (RA) with biological disease-modifying anti-rheumatic drugs (bDMARDs) induces rapid remission. However, osteoporosis and its management remains a problem. The Geriatric Nutritional Risk Index (GNRI) evaluates the risk of malnutrition-related complications in elderly patients and has been shown to be a significant predictor of many diseases. We evaluated the correlation between GNRI and RA activity. In addition, risk factors for femoral neck bone loss were evaluated in RA patients treated with bDMARDs. We retrospectively examined the medical records of 146 patients with RA, collecting and recording the patients’ demographic and clinical characteristics. Bone mineral density (BMD) was measured by dual-energy X-ray absorptiometry. Inverse correlations were observed between GNRI and disease duration, disease activity score-28 joint count serum C-reactive protein (CRP), simple disease activity index, modified health assessment questionnaire score and CRP. GNRI showed correlation with femoral neck BMD and femoral neck BMD ≤ 70% of young adult men (YAM). Multiple regression analysis showed that female sex, increased age and lower GNRI were risk factors for lower BMD of the femoral neck. Multivariate binomial logistic regression analysis showed that female sex (odd ratio: 3.67) and lower GNRI (odd ratio: 0.87) were risk factors for BMD ≤ 70% of YAM. Because the GNRI is a simple method, it might be a simple predictor for RA activity and BMD status in RA patients. Complementary nutritional therapies might improve RA activity and osteoporosis in RA patients who have undergone treatment with bDMARDs.

## 1. Introduction

Rheumatoid arthritis (RA) is a chronic, systemic, inflammatory disorder that promotes joint inflammation and destruction [1]. Treatment options for RA have increased rapidly using biological disease-modifying anti-rheumatic drugs (bDMARDs) [2]. bDMARDs include tumor necrosis factor-α inhibitors, anti-interleukin-6 agents, B cell-depleting agents and cytotoxic T lymphocyte-associated antigen 4 therapy. Treatment with bDMARDs induces rapid accomplishment of remission, with reduced radiological progression and disability [3,4]. However, co-morbidities remain a problem in RA patients that must be managed [5]. RA increases the risk of bone loss and fracture, which are associated with a reduction in quality of life [5]. Poor nutrient status in RA patients is often reported [6], therefore nutrient supplementation should be considered in the management of RA and its co-morbidities [7]. It has been reported that poor nutritional status was related to osteoporosis in postmenopausal women [8,9]. Nutrients, including micronutrients—calcium, vitamin D, phosphorous and magnesium—are key constituents of bone. However, protein is an often-neglected bone constituent and contributor to bone health [10,11]. Large-scale literature reviews showed that increased protein intake in the diet is beneficial to bone health and reduces morbidity and mortality [12]. The Geriatric Nutritional Risk Index (GNRI) is advocated to evaluate the risk of malnutrition-related complications in elderly patients [13]. The GNRI has been reported to be a significant predictor of prognosis in hemodialysis [14,15,16,17], percutaneous coronary intervention [18], systolic heart failure [19], pneumonia [20], diabetic foot ulcers [21], a predictor of prevalence coronary artery [22], acute heart failure [23], squamous cell carcinoma [24] and obstructive pulmonary disease patients [25]. We evaluated the correlation between GNRI and disease activity in the RA patients treated with bDMARDs. In addition, risk factors for femoral neck bone loss were evaluated in the RA patients treated with bDMARDs.

## 2. Subjects and Methods

### 2.1. Participants

We retrospectively examined the records of 146 patients with RA, diagnosed using the 2010 criteria of the American College of Rheumatology and treated with infliximab, adalimumab, golimumab, etanercept, tocilizumab or abatacept at the Japanese Red Cross Kagoshima Hospital.

### 2.2. Demographic and Disease-Related Data

Patients’ demographic and clinical characteristics were collected from medical records, including sex, age, disease duration, estimated glomerular filtration rate (eGFR) (mL/min/1.73 m^3^), dose of corticosteroid (mg), GNRI, disease activity score-28-reactive protein (DAS28-CRP), Simplified Disease Activity Index (SDAI), modified health assessment questionnaire (MHAQ) score, serum C-reactive protein (CRP) (mg/dL) and bone mineral density (BMD) of the femoral neck (g/cm^2^). The patients for whom some of these data were missing were excluded from the study.

### 2.3. Dual Energy X-ray Absorptiometry Measurements

BMD was examined between December 2011 and December 2013 using the Discovery DXA system (Hologic, Waltham, MA, USA). The Japanese Society for Bone and Mineral Research has proposed that primary osteoporosis is diagnosed when BMD is ≤ 70% in young adult men (YAM) [26,27,28,29]. We therefore separated the patients into two groups: those with BMD ≤ 70% and those with BMD > 70%.

### 2.4. Geriatric Nutritional Risk Index

The GNRI was calculated as previously reported [13]. GNRI was derived from serum albumin and body weight as described in Equation (1), A represents the albumin, the unit is g/L; B represents body weight; C represents ideal body weight:

GNRI = 1.489 A + 41.7 B/C(1)

In Equation (1), A represents the albumin, the unit is g/L; B represents body weight; C represents ideal body weight.

Body weight or ideal body weight were set to 1 when the patient’s body weight exceeded the ideal body weight. The ideal body weight was defined as a BMI of 22 [14,30].

### 2.5. Statistical Analysis

The Kolmogorov–Smirnov test was used to evaluate the distribution of data. The data were examined using analysis of variance (ANOVA), Kruskal–Wallis analysis, or Student’s *t*-test. Spearman’s rank correlation coefficient analysis was performed for the correlation of variables. Multivariable logistic regression analysis and multivariate binomial logistic regression analysis were performed to correlate demographic data with BMD. All variables were included in the multivariable logistic regression analysis and multivariate binomial logistic regression analysis. Analysis was performed using BellCurve for Excel (Social Survey Research Information Co., Ltd., Tokyo, Japan).

### 2.6. Ethics Approval and Consent to Participate

This research protocol was approved by the Ethics Committee on Clinical Research at the Japanese Red Cross Kagoshima Hospital (Approval No. 2016-1-1). This research was executed in accordance with the Helsinki Declaration of 1975, as revised in 2008. All subjects gave their informed consent for inclusion before they participated in the study.

## 3. Results

### 3.1. Relationship between GNRI, Disease Activity and BMD

**Figure 1:**
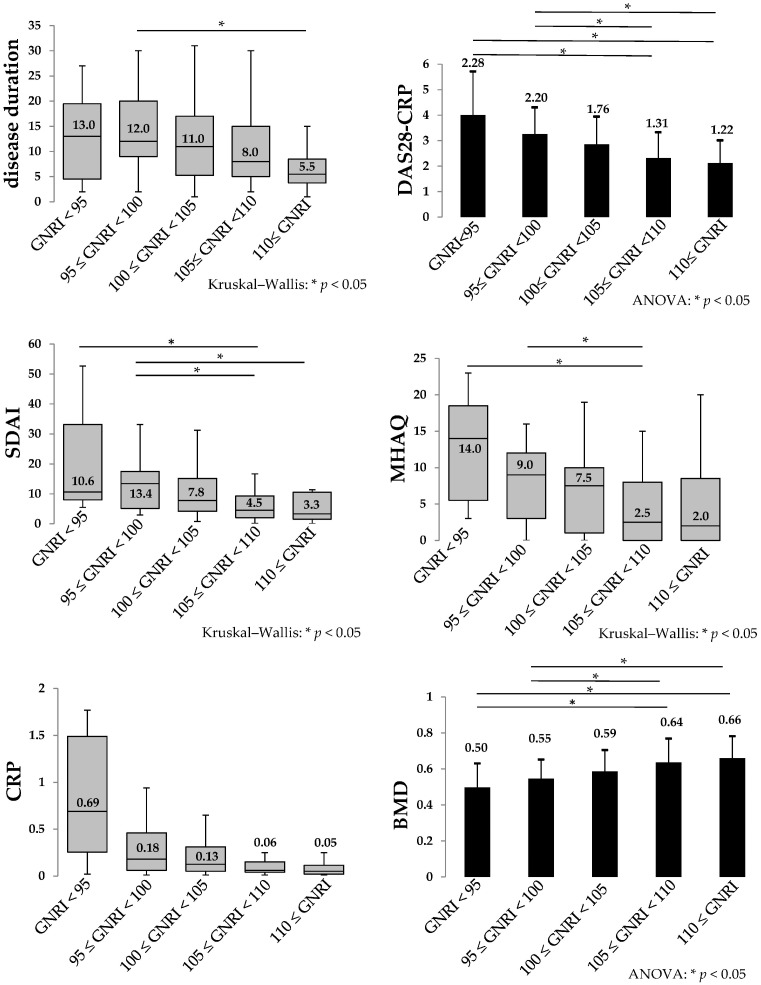
Correlation between the Geriatric Nutritional Risk Index (GNRI) and rheumatoid arthritis (RA) disease activity and bone mineral density (BMD). Patients were divided into 5 groups: (1) GNRI < 95 (*n* = 7); (2) GNRI ≥ 95 but < 100 (*n* = 21); (3) GNRI ≥ 100 but < 105 (*n* = 42); (4) GNRI ≥ 105 but < 110 (*n* = 56); (5) GNRI ≥ 110 (*n* = 20). The Kolmogorov–Smirnov test was used to evaluate the distribution of the data. The data were then examined using analysis of variance or the Kruskal–Wallis analysis. Disease duration, Disease Activity Score-28-C-reactive protein (DAS28-CRP), Simplified Disease Activity Index (SDAI), Modified Health Assessment Questionnaire (MHAQ) and bone mineral density (BMD) showed statistically significant differences between groups (*p* < 0.05). AVONA: Analysis of variance.

**Figure 2:**
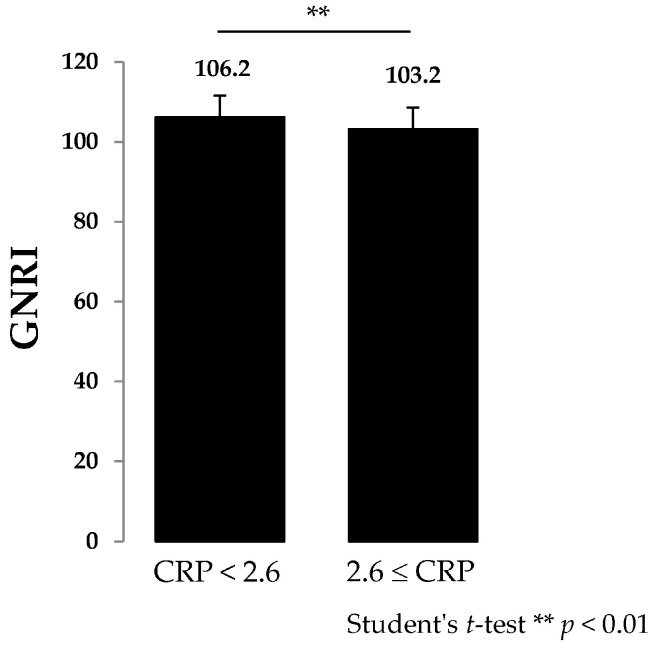
Difference in GNRI between the CRP < 2.6 and ≥ 2.6 groups. Kolmogorov–Smirnov test showed that data were in normal distribution. Student’s *t*-test showed a statistically significant difference between the two groups (*p* < 0.05).

The demographic and clinical characteristics of the 146 patients who underwent DXA scanning of the femoral neck are shown in Table 1. All variables were evaluated by Spearman’s rank correlation coefficient analysis. Weak inverse correlation between GNRI and disease duration (*r*: −0.27), DAS28-CRP (*r*: −0.39), SDAI (*r*: −0.34), MHAQ (*r*: −0.33) and CRP (*r*: −0.25) were observed (Table 2). In addition, GNRI showed weak correlation with femoral neck BMD (*r*: 0.35) and femoral neck BMD ≤ 70% of YAM (*r*: −0.34) (Table 2). Patients were divided into 5 groups: (1) GNRI < 95 (*n* = 7); (2) GNRI ≥ 95 but < 100 (*n* = 21); (3) GNRI ≥ 100 but < 105 (*n* = 42); (4) GNRI ≥ 105 but < 110 (*n* = 56); (5) GNRI ≥ 110 (*n* = 20). DAS28-CRP and BMD showed statistically significant differences between groups (1) and (2) and groups (4) and (5) (Figure 1). Next, patients were divided into 2 groups (1) DAS28-CRP < 2.6 (*n* = 78) and DAS28-CRP ≥ 2.6 (*n* = 68) according to a previous report [31]. GNRI of the DAS28-CRP < 2.6 group was significantly lower than that of DAS28-CRP ≥ 2.6 (Figure 2).

### 3.2. Low GNRI Is a Risk Factor for Low BMD of the Femoral Neck

Multiple regression analysis was performed to evaluate risk factors for lower BMD of the femoral neck in the RA patients treated with bDMARDs. Female sex, older age and lower GNRI were identified as risk factors for lower BMD of the femoral neck (Table 3). Furthermore, higher age and lower GNRI were identified as risk factors for lower BMD of YAM in the femoral neck (Table 4). Multivariate binomial logistic regression analysis showed that female sex (odds ratio: 3.67) and lower GNRI (odds ratio: 0.87) were risk factors for BMD ≤ 70% in YAM (Table 5).

## 4. Discussion

The GNRI was initially developed to evaluate the risk of malnutrition-related complications in elderly patients [13] and has since been shown to be a significant predictor of many diseases [14,15,16,17,18,19,20,21,22,23,24]. We therefore used GNRI in our examinations and showed that GNRI inverse correlated with DAS28-CRP, SDAI and CRP. To our knowledge, this is the first report showing the relationship between GNRI and disease activity of RA. Because GNRI is a simple method to evaluate nutritional status using just blood albumin, body weight and height, GNRI is a simple and useful tool to evaluate the nutritional status and disease activity in RA patients and that improvement of nutrition might have a positive effect in decreasing disease activity.

It is reported that 33–75% of RA patients believe that food plays an important role in the severity of their disease activity and that 20–50% of RA patients will have tried dietary manipulation in an attempt to decrease pain [32,33]. Nutritional status of RA patients is compromised despite relevant ingestion. Rheumatoid cachexia, a condition of up-regulated metabolism that leads to reduction of weight and body mass, requires that attention be paid to protein and energy balance [34]. Our findings showed that low GNRI is a significant risk factor for lower BMD of the femoral neck in RA patients, even when treated with bDMARDs. To our knowledge, this is the first report to show the direct relationship between GNRI and BMD. GNRI might be a simple predictor for the BMD status in RA patients. The average GNRI of these patients was 104.8 ± 5.5, which is not a severe nutritional status. When we divided patients in 5 groups according to the GNRI, however, we found that lower GNRI is correlated with higher DAS28-CRP, higher SDAI, higher MHAQ and lower BMD. These findings partially validate that improved nutrition is related to lower levels of disease activity and lower BMD in patients with a low GNRI.

The relationship between nutritional status and bone mass in chronic obstructive pulmonary disease [35], chronic kidney disease [36], non-alcoholic fatty liver disease [37] and cardiovascular disease [38] has been established. Therefore, GNRI might be a simple predictor for BMD status in patients with these diseases. It has been reported that significant negative association between protein consumption and risk of hip fracture [39,40,41]. Because both animal and vegetable sources of protein contributed to this association [39], it is important to take sufficient proteins from these diets.

It has been reported that DAS28-CRP < 2.6 indicated remission [31]. Although 78 patients were in the DAS28-CRP < 2.6 group, 27 of 78 (34.6%) patients had a BMD ≤ 70% in the femoral necks of YAM. These findings suggest that early prevention and treatment of osteoporosis might be needed for RA patients treated with bDMARDs.

Our study has several limitations. First, this study was a cohort single-center study, so selection bias may have occurred. In future, a multicenter study should be performed to confirm our findings. Second, because this study evaluated single-time-point data, a longitudinal study is needed to evaluate changes in each factor. Consequently, increases in the number of variables and patients are needed to identify important and precise risk factors in future studies. We did not evaluate the diets of patients who were unrelated to the medical institution. More detailed interviews of patients’ dietary habits should be performed. Associations between vitamin D deficiency and poor bone health were reported [42,43]. Because vitamin D deficiency is highly prevalent in RA patients [44], the relationship between vitamin D status and osteoporosis should be examined in future studies.

## 5. Conclusions

Our findings suggest that GNRI might be a simple predictor of RA activity and BMD of the femoral neck. Complementary nutritional therapies might improve RA activity and osteoporosis in RA patients treated with bDMARDs.

## Figures and Tables

**Table 1:** Patients’ demographic data.

Variables	
Female proportion	82.9%
Age (years)	60.5 ± 10.3
Disease duration (years)	9.5 (5–16)
eGFR (mL/min/1.73 m^3^)	74.4 ± 18.4
Dose of corticosteroid (mg)	0.0 (0.0–1.9)
GNRI	104.8 ± 5.5
DAS28-CRP	2.5 (1.7–3.3)
SDAI	6.2 (2.9–12.8)
MHAQ	4.0 (0.0–10.0)
CRP (mg/dL)	0.09 (0.03–0.31)
Femoral neck BMD (g/cm^2^)	0.61 ± 0.13
Proportion of BMD ≤ 70% of YAM	38.4%

Abbreviations: eGFR, estimated glomerular filtration rate; GNRI, geriatric nutritional risk index; DAS28-CRP, Disease Activity Score-28-CRP; SDAI, Simplified Disease Activity Index; MHAQ, Modified Health Assessment Questionnaire; CRP, serum C-reactive protein concentration; BMD, bone mineral density; YAM, young adult mean.

**Table 2:** Correlation between GNRI and other variables.

Variables	Correlation Coefficient
	GNRI
Sex	−0.09
Age	−0.08
Disease duration	−0.27 ***
eGFR (mL/min/1.73 m^3^)	−0.06
Dose of corticosteroid (mg)	−0.19 *
DAS28-CRP	−0.39 ***
SDAI	-0.34 ***
MHAQ	−0.33 ***
CRP (mg/dL)	−0.25 **
Femoral neck BMD (g/cm^2^)	0.35 ***
Femoral neck BMD ≤ 70% of YAM	0.34 ***

* *p* < 0.05; ** *p* < 0.01; *** *p* < 0.001. Abbreviations: eGFR, estimated glomerular filtration rate; GNRI, geriatric nutritional risk index; DAS28-CRP, Disease Activity Score-28-CRP; SDAI, Simplified Disease Activity Index; MHAQ, Modified Health Assessment Questionnaire; CRP, serum C-reactive protein concentration; BMD, bone mineral density; YAM, young adult men.

**Table 3:** Risk factors for lower BMD in the femoral neck.

Variables	Partial Regression Coefficient	*p*-Value
Sex	−0.106	<0.001
Age	−0.003	0.003
Disease duration	−0.002	0.175
eGFR (mL/min/1.73 m^3^)	−0.001	0.202
Dose of corticosteroid (mg)	−0.003	0.584
DAS28-CRP	0.024	0.307
SDAI	−0.001	0.750
MHAQ	−0.004	0.098
CRP (mg/dL)	0.007	0.677
GNRI	0.007	<0.001

Abbreviations: eGFR, estimated glomerular filtration rate; GNRI, geriatric nutritional risk index; DAS28-CRP, Disease Activity Score-28-CRP; SDAI, Simplified Disease Activity Index; MHAQ, Modified Health Assessment Questionnaire; CRP, serum C-reactive protein concentration.

**Table 4:** Risk factors for lower BMD of YAM in the femoral neck.

Variables	Partial Regression Coefficient	*p*-Value
Sex	−6.050	0.052
Age	−0.426	0.002
Disease duration	−0.196	0.252
eGFR (ml/min/1.73 m^3^)	−0.095	0.201
Dose of corticosteroid (mg)	−0.332	0.604
DAS28-CRP	2.622	0.360
SDAI	−0.136	0.694
MHAQ	−0.425	0.117
CRP (mg/dL)	1.001	0.613
GNRI	0.792	0.001

Abbreviations: eGFR, estimated glomerular filtration rate; GNRI, geriatric nutritional risk index; DAS28-CRP, Disease Activity Score-28-CRP; SDAI, Simplified Disease Activity Index; MHAQ, Modified Health Assessment Questionnaire; CRP, serum C-reactive protein concentration.

**Table 5:** Risk factors for BMD ≤ 70% in the femoral necks of YAM.

Variables	Odds Ratio	*p*-Value
Sex	3.67 (1.06–12.9)	0.040
Age	1.04 (0.99–1.09)	0.115
Disease duration	1.05 (0.99–1.11)	0.101
eGFR (mL/min/1.73 m^3^)	1.01 (0.99–1.04)	0.378
Dose of corticosteroid (mg)	0.84 (0.66–1.06)	0.147
DAS28-CRP	0.81 (0.30–2.15)	0.666
SDAI	1.01 (0.90–1.13)	0.874
MHAQ	1.06 (0.97–1.16)	0.117
CRP (mg/dL)	1.19 (0.61–2.32)	0.612
GNRI	0.87 (0.80–0.95)	0.002

Abbreviations: eGFR, estimated glomerular filtration rate; GNRI, geriatric nutritional risk index; DAS28-CRP, Disease Activity Score-28-CRP; SDAI, Simplified Disease Activity Index; MHAQ, Modified Health Assessment Questionnaire; CRP, serum C-reactive protein concentration.
